# A Polymethionine Nanoparticle Fluorescent Probe for Sensitive Detection of Naringin and Naringenin

**DOI:** 10.3390/ma17163919

**Published:** 2024-08-07

**Authors:** Yuhong Jiao, Lu Li, Jinlong Ge, Yanfang Tai, Hui Han

**Affiliations:** 1 School of Materials and Chemical Engineering, Bengbu University, Bengbu 233000, China; jwh@bbc.edu.cn (Y.J.); jinlongge2005@126.com (J.G.); taiyanfang@163.com (Y.T.); 2 Anhui Triumph Applied Materials Co., Ltd., Bengbu 233000, China; 13956386350@163.com

**Keywords:** fluorescence, polymethionine nanoparticles, naringin and naringenin detection, inner filter effect

## Abstract

In this work, we demonstrated a novel, sensitive and effective fluorescent naringin (NRG) and naringenin (NRGe) detection method using polymethionine nanoparticles (PMNPs) as a fluorescent nanoprobe. The PMNPs were first synthesized by autopolymerization of methionine at 90 °C when trace copper ions existed. The as-prepared PMNPs were thoroughly characterized by transmission electron microscopy (TEM), Fourier-transform infrared spectroscopy (FT-IR), gel permeation chromatograph (GPC), nuclear magnetic resonance spectroscopy (NMR), transient and steady-state fluorescence and UV–Vis absorption spectroscopy. The quenching mechanism was attributed to the inner filter effect (IFE). Moreover, the developed assay was used successfully to detect NRG and NRGe in real samples of citrus fruits, illustrating that this detection method has great potential application in the field of citrus fruits analysis.

## 1. Introduction

Flavonoids are polyphenolic compounds consisting of three phenolic rings that are widely present in many citrus fruits, as well as in various plants and traditional Chinese medicinal herbs [1,2,3]. Among several varieties of flavonoids, naringin (NRG) and naringenin (NRGe) are considered an important class of flavonoids with a wide range of physiological functions such as anti-inflammatory [4], antioxidation [5], anti-ulcer and anti-cancer activities [6,7]. NRG (4,5,7-trihydroxy flavanone-7-rhamnongluco-side) is a dihydroflavonoid compound with various biological activities and pharmacological effects. It can lower blood cholesterol, reduce thrombosis formation and improve local microcirculation and nutritional supply, which can be used for production prevention and the treatment of cardiovascular and cerebrovascular diseases [8,9]. NRGe (4,5,7-trihydroxyflavanone) is the aglycone of naringin, a dihydroflavonoid compound. It has desirable properties such as antibacterial [10,11], antitussive and expectorant [12], anti-cancer [13,14] and antiatherosclerosis properties [15] and can be widely used in medicine, food and other fields. Hence, the detection of NRG and NRGe in citrus fruits and biological systems is of great importance and significance.

So far, a few methods have been developed for NRG and NRGe detection, mainly including high-performance liquid chromatography (HPLC) [16,17], gradient elution liquid chromatography–selected ion monitoring mass spectrometry (SIM LC–MS) [18,19] and electrochemical methods [20,21,22,23,24,25]. As we know, HPLC and SIM LC–MS have some drawbacks such as the requirement for expensive instrumentation, being time-consuming and implying complicated operations. Therefore, the development of a simple, rapid, sensitive and effective assay for detecting naringin and naringenin in food samples is still desired. Compared to HPLC and SIM LC–MS, electrochemical methods are simpler and cost less and have been increasingly favored. For example, Camila S. Sousa et al. developed a photoelectrochemical sensor for the determination of naringin on the basis of a modified FTO electrode with cadmium sulfide and titanium dioxide sensitized with chloroprotoporphyrin IX iron (III) [20]. Ziyatdinova developed an electrochemical sensor using a glassy carbon electrode (GCE) modified with multi-walled carbon nanotubes and electropolymerized ellagic acid (polyEA/MWNT/GCE) for naringin sensing [21].

**Scheme 1:**
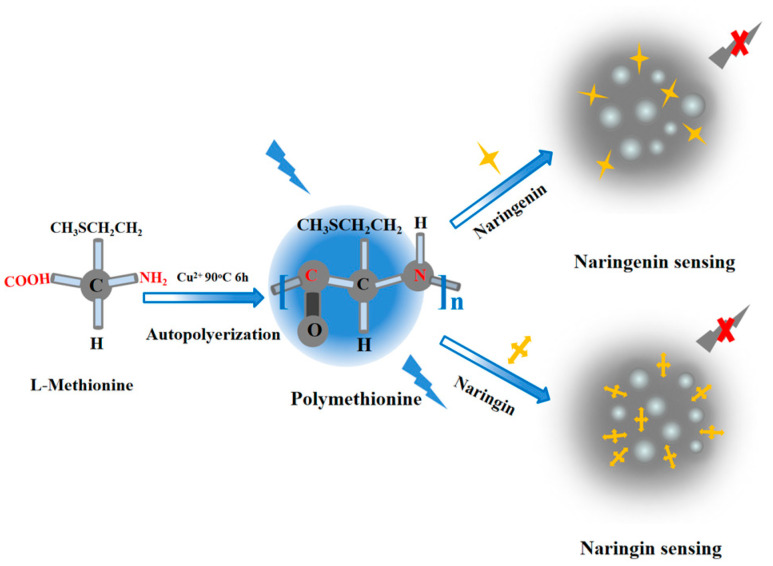
The illustration of the synthesis of PMNPs as a fluorescent assay for naringenin and naringin sensing.

However, to the best of our knowledge, fluorescent methods have never been used in NRG and NRGe detection. As it is known, fluorescent methods possess the advantages of simplicity and portability as well as sensitive detection of analytes in food, cells and in vivo. Fluorescent probes are equally fascinating. For example, Çenet developed a fluorescent probe for fenamiphos detection in not only vegetables but also living cells [26]. Jiang designed a pyrene-based fluorescent probe for the detection of hypochlorite in aqueous solutions and in living cells [27]. Previously, Su reported that polydihydroxypheny-lalanine nanoparticles (PDNPs) were prepared for Cu^2+^ sensing [28]. Yildirim reported on polydopamine nanoparticles for dopamine detection [29]. In the present work, we first prepared fluorescent polymethionine nanoparticles (PMNPs) with a methionine solution under alkaline conditions at 90 °C in the presence of copper ions. When NRG/NRGe was introduced into the PMNP solution, the fluorescent intensities decreased, which was ascribed to the inner filter effect (IFE) (Scheme 1).

Herein, this work presents the first sensing platform with a fluorescent probe for the sensing of NRG and NRGe, which provides a novel perspective on the detection of NRG/NRGe. The strategy is simple, economical and sensitive. The detection limit of NGR was 0.33 μΜ and NRGe was 0.83 μΜ, which is comparable to some reported electrical methods (Table 1 and Table 2). Moreover, the as-prepared PMNPs have been successfully applied in the detection of NRG and NRGe in real samples of citrus fruits. The probe possesses good stability and water solubility and therefore has immense potential in the further detection of NRG/NRGe in living cells and in vivo.

## 2. Experimental Section

### 2.1. Materials

L-methionine (L-Met) was purchased from Shanghai Sangon Biotech Co., Ltd. (Shanghai, China). Anhydrous copper sulfate (CuSO_4_) and sodium hydroxide (NaOH) were obtained from Shanghai Sinopharm Chemical Reagent Co., Ltd., Shanghai, China. Naringin and naringenin were purchased from Aladdin scientific Co., Ltd. (Shanghai, China). Other chemicals of analytical purity were obtained from Sinopharm Chemical Reagent Co., Ltd. (Shanghai, China), including MgSO_4_, HgNO_3_, NaCl, CoCl_2_, KIO_3_, Fe_2_(SO_4_)_3_, KI, Al_2_(SO_4_)_3_, (NH_4_)_2_S_2_O_8_, KCl, CdCl_2_, CaCl_2_, BaCl_2_, Na_2_S, K_4_O_7_P_2_ (PPi), histidine (His), tyrosine (Tyr), glutathione (Glu), urea, uric acid (UA), dopamine (DA), methoxymethyldiphenylamine (DPA) and H_2_O_2_. The water we used throughout the process was double-deionized water (DDW). Robinson buffer solution (BR buffer, H_3_PO_4_-HAc-H_3_BO_3_, 0.04 M) was prepared, and its pH value was adjusted using 0.2 M NaOH solution.

### 2.2. Synthesis of the PMNPs

Firstly, 0.5 mL of 2.5 mM CuSO_4_ was added into 25 mg/mL of L-methionine solution (0.4 M NaOH). The solution was continuously stirred for 6 h at 90 °C. The mixture eventually turned light yellow, which indicates that the PMNPs have formed. Ultimately, the mixture was treated via 12 h dialysis with ultrapure water, the resultant solution of PMNPs was stored in a refrigerator at 4 °C until further use (Scheme 1).

### 2.3. Characterizations

Fluorescence was measured using an FP-8300 spectrophotometer (Jasco, Tokyo, Japan). An absorption measurement was performed using a PGENERAL TU-1901 UV–vis spectrophotometer (Beijing Purkinje general instrument Co. Ltd. China). Fourier-transform infrared (FTIR) spectroscopy was investigated using a */NEXUS470 (Thermo Fisher, Waltham, MA, USA). Transmission electron microscopy (TEM) was characterized using a Jeol Jem 2100 microscope (JEOL, Tokyo, Japan). Fluorescence lifetime experiments were recorded by a FLS920 fluorescence spectrometer (Edinburgh Instrument, Livingston, UK). Gel permeation chromatography was performed using an Agilent 1260 Infinity II GPC spectrometer (Polytech, Beijing, China). ^1^H NMR imaging was performed using a Bruker Avance Neo 600M NMR spectrometer (Bruker, Fällanden, Switzerland).

### 2.4. General Procedure for NRG and NRGe Sensing

Firstly, 200 μL of PMNPs solution was diluted 10 times using DDW. Then, 100 μL of different concentrations of NRG were added to the diluted assay solution and the final concentrations of the NRG solution were 0, 1, 2, 3, 4, 5, 10, 15, 20, 25, 30, 40, 50, 60, 70, 80, 90, 100, 125, 150 and 200 μΜ. The mixture was shaken thoroughly at room temperature.

The detection steps for NRGe are the same as the above steps. The final concentrations of the NRGe solution were 0, 2.5, 5, 10, 15, 20, 25, 30, 35, 40, 45, 50, 60, 70, 80, 90, 100, 150 and 200 μΜ. The fluorescence intensity of all the samples was monitored immediately under excitation at 370 nm.

### 2.5. Analysis of NRG and NRGe in Citrus Fruits Samples

Fluorescence assay of NRG and NRGe in real samples (grapefruit peel and orange juice) was performed using PMNPs as a probe. The grapefruit and orange were purchased from the local supermarket. Firstly, grapefruit peel was weighed and crushed into a powder. Then, the powder was dissolved in sodium hydroxide solution (0.4 M) and filtered. The filtrate was diluted using ultrapure water to form an ultima solution. Next, 100 μL of the diluted solution extracted from the grapefruit peel was spiked with different amounts of NRG (final concentrations: 2.5, 5, 10 μΜ) and NRGe (final concentrations: 5, 10, 20 μΜ), respectively, which were used for further analysis using the above-mentioned procedure.

The orange flesh and an amount of water was added into the juicer. After crushing, the resultant solution was centrifuged at 12,000 rpm for 10 min and the supernatant was collected for further use. Next, 100 μL of supernatant was spiked with different amounts of NRG (final concentrations: 2.5, 5, 10 μΜ) and NRGe (final concentrations: 5, 10, 20 μΜ), respectively, which were used for further analysis using the above-mentioned procedure.

## 3. Results and Discussion

### 3.1. Characterization of PMNPs

**Figure 1:**
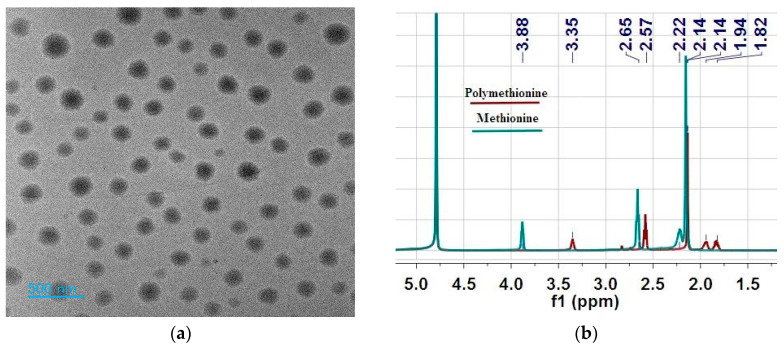

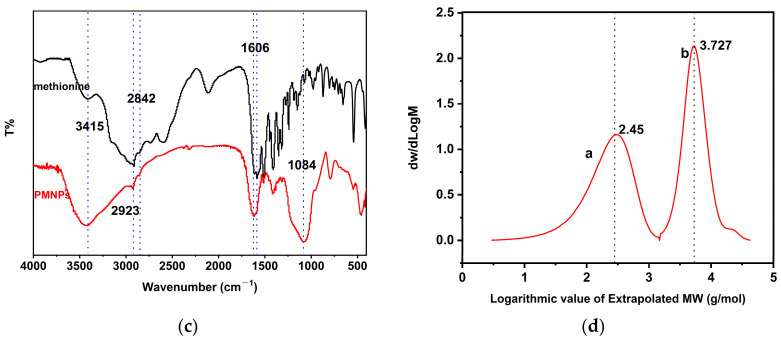
(**a**) TEM image of the PMNPs. (**b**) ^1^H NMR image of methionine (green line) and PMNPs (red line). (**c**) FTIR-spectra of L-methionine (black line) and PMNPs (red line). (**d**) GPC survey spectrum of methionine (**a**) and PMNPs (**b**).

Blue fluorescent PMNPs were developed using a “one-pot” strategy, in which L-Mets self-polymerize into oligomers with fluorescent properties under the catalytic action of copper ions (Scheme 1). The synthesized PMNPs were characterized using various techniques. In order to demonstrate the formation of PMNPs, TEM analysis was performed. The TEM image showed that the obtained PMNPs were spherical in shape and had a uniform size distribution (Figure 1a). The average diameter of the PMNPS was determined to be 157.08 ± 31.83 nm (Figure S1), which suggested the formation of homogeneous small PMNPs. To verify the formation of the PMNPs, ^1^H NMR analysis was performed. The ^1^H NMR image showed that the PMNPs had a typical amide bond, indicating that autopolymerization had occurred (Figure 1b). The surface groups of methionine and PMNPs were analyzed using FT-IR spectra. Figure 1c shows that methionine and the PMNPs had similar peak positions and main functional groups, indicating that methionine was successfully autopolymerized. The transmission peaks at 1606 cm^−1^ suggested a correspondence to the C=O stretching vibration. The peak at 3406 cm^−1^ was broad, which corresponded to the stretching of the hydroxyl group. The GPC spectra was used to analyze the molecular weight of PMNPs. Figure 1d shows that the PMNPs had a molecular weight of over 5000.

**Figure 2:**
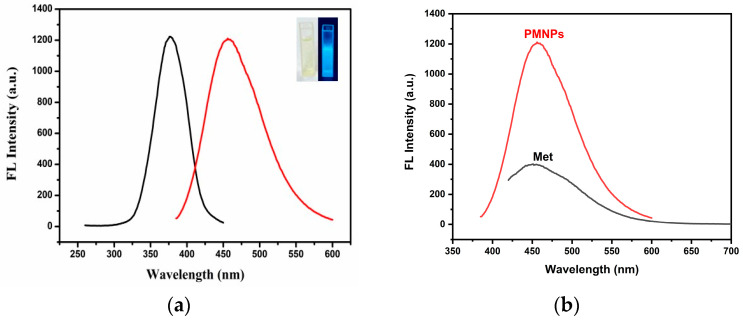

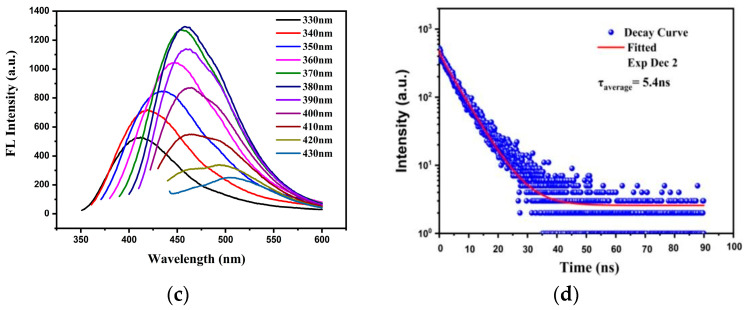
(**a**) FL excitation (black line) and emission (red line) spectra of PMNPs. Inset: the photo of the prepared PMNPs under daylight (left) and 365 nm UV light (right). (**b**) Fluorescence emission spectra of Met (black line) and PMNPs (red line). (**c**) Fluorescence emission spectra of PMNPs with various excitation wavelengths from 330 to 430 nm. (**d**) Time-resolved fluorescence spectra of PMNPs collected at 375 nm excited.

The fluorescence spectra of the PMNPs are shown in Figure 2. The excitation of the PMNPs was 377 nm and emissions were 456 nm. The as-synthesized PMNPs solution was light yellow in daylight and emitted a strong blue fluorescence under 365 nm UV light irradiation (inset Figure 2a). In Figure 2b, L-Met had a weak fluorescence intensity, whereas L-Met with trace copper ions showed a strong fluorescence peak, resulting from methionine undergoing self-polymerization. As shown in Figure 2c, when the excitation wavelengths changed from 330 nm to 430 nm, the fluorescence intensity of PMNPs increased and then decreased, indicating the excitation-dependent emission behavior of the resulting PMNPs, which was very similar to eumelanin polymers [30]. PMNPs displayed a broad absorbance around 273 nm (Figure S2). The characteristic absorption peak at 560–600 nm, arising from the surface plasmonic resonance (SPR) of large-sized Cu nanoparticles, was not observed, demonstrating the formation of PMNPs rather than metal cousin nanoparticles [31,32,33]. Additionally, the time-resolved FL decay of PMNPs was further investigated using the time-correlated single-photon counting technique (TCSPC) measurement (Figure 2d). The FL decay curve could be well-fitted by a two-exponential function: I(t) = B_1_exp(−t/τ_1_) + B_2_exp(−t/τ_2_), where τ_1_ and τ_2_ represent the decay time, and B_1_ and B_2_ are the corresponding amplitudes. Fluorescence decay was calculated according to the following equation: τaverage = (B_1_τ_1_^2^ + B_2_τ_2_^2^)/(B_1_τ_1_ + B_2_τ_2_) [34]. The average FL lifetime was calculated to be 5.4 ns.

### 3.2. Stability Studies of PMNPs

**Figure 3:**
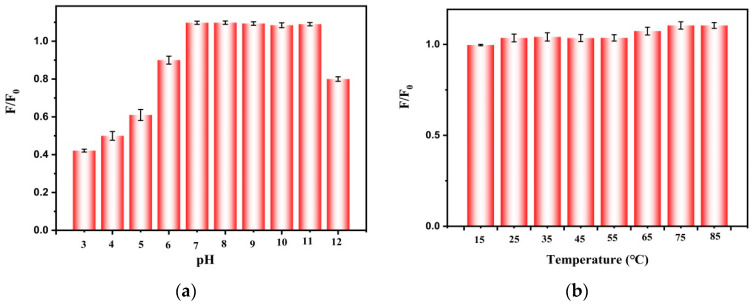
(**a**) The relative fluorescence intensity (F/F_0_) of the PMNPs in BR buffer at various pH values in a range from 3 to 12. (**b**) The relative fluorescence intensity (F/F_0_) of the PMNPs at different temperatures ranging from 25 to 85 ^◦^C. F_0_ refers the fluorescence intensity of the PMNPs at room temperature and F refers the fluorescence intensity of the PMNPs at different temperatures.

The stability of PMNPs is vital for their potential applications. The tolerance of the prepared PMNPs to pH and temperature was investigated and is shown in Figure 3a. The fluorescence value of the PMNPs decreased in strongly acidic media and was relatively stable in alkaline, neutral and weakly acidic media. Amidogen reacts with carboxyl groups to form ester groups in the process of self-polymerization of L-methionine. The ester group is completely hydrolyzed under acidic conditions, inhibiting the self-polymerization of L-methionine, possibly resulting in the significant decreases in fluorescence value. Although the hydrolysis of the ester group is reversible under alkaline conditions, the PMNPs were stable in alkaline, neutral and weakly acidic conditions. A possible reason for this is that the hydrolysis of the ester groups under alkaline and weakly acidic conditions is a dynamic equilibrium process, which does not destroy methionine autopolymerization. Moreover, no hydrolysis of the ester groups took place under neutral conditions. Figure 3b shows that the fluorescence intensity of PMNPs in the temperature range of 15–85 °C showed little change, indicating that the prepared PMNPs had good temperature resistance.

### 3.3. Fluorescence Sensing of NRG and NRGe Based on the PMNPs Probe

**Figure 4:**
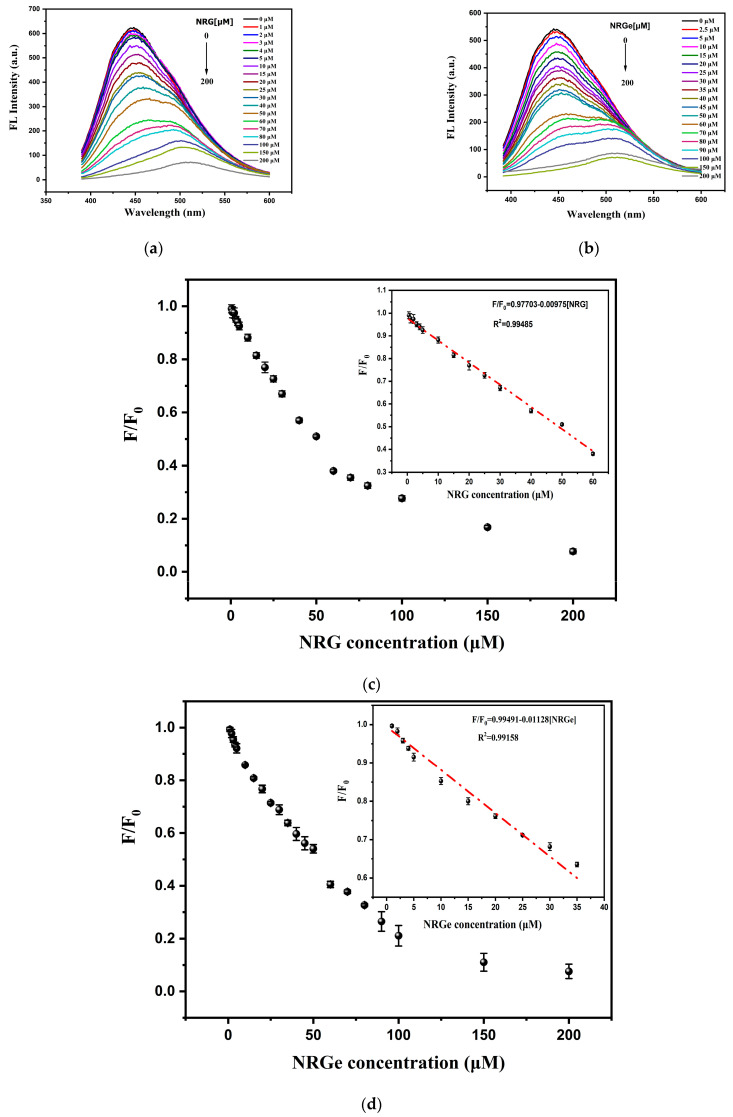
Fluorescence emission spectra of PMNPs with various concentrations (0–200 μM) of NRG (**a**) and NRGe (**b**). The plot of relative emission intensity (F/F_0_) versus different concentrations of NRG (**c**) and NRGe (**d**). Inset: Relative emission intensity (F/F_0_) of PMNPs versus the NRG (**c**) and NRGe (**d**) concentrations from 0 to 60 μM and 0 to 35 μM, respectively. F_0_ and F refer to the fluorescence intensity of PMNPs solution in the absence and presence of NRG/NRGe, respectively.

As shown in Figure 4a,b, PMNPs exhibited a strong emission intensity in the absence of NRG/NRGe, whereas the fluorescent values of PMNPs gradually dropped as the concentrations of NRG/NRGe increased. It is noted that the PMNPs were quenched linearly upon the addition of NRG/NRGe in the concentration range of 0~200 μΜ (Figure 4c,d). There was a positive linear relationship between the relative fluorescence intensity of the PMNPs versus NRG/NRGe concentrations in a range between 0.00~60.00 μM and 0.00~35.00 μM, with correlation coefficients of 0.99485 and 0.9915, respectively (inset in Figure 4c,d). The linear equation of NRG and NRGe is F/F_0_ = 0.97703 − 0.00975 × C[NRG] and F/F_0_ = 0.99491 − 0.01128 × C[NRGe], where F_0_ and F refer to the fluorescence intensity of the PMNPs solution in the absence and presence of NRG/NRGe, respectively. The limit of detection (LOD) was calculated from the following relationship: S/N = 3, where N is the standard deviation of the blank signal and S is the slope of the linear calibration plot. The computed LOD of NRG was 0.33 μM and that of the NRGe was as low as 0.83 μM. In comparison with other methods such as MLCEC and HPLC-MS, the fluorophotometric method was cost-effective and simple and its LOD was comparable to other reported methods (Table 1 and Table 2).

### 3.4. Selectivity of the Sensing System

**Figure 5:**
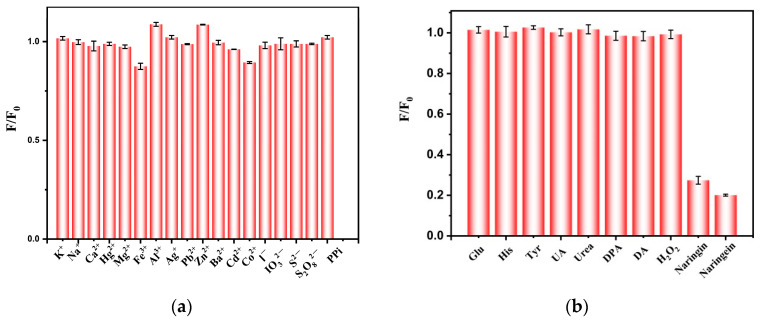
Selectivity of fluorescent PMNPs probe towards some molecules (**a**), cations, and anions (**b**). F_0_ and F are the fluorescence intensity of PMNPs in the absence and presence of different species, respectively.

In order to assess the selectivity of the PMNPs assay in NRG and NRGe sensing, we examined the fluorescent intensity change of the assay in the presence of 100 μM of various common molecules such as histidine(His), tyrosine (Tyr), glutathione (Glu), urea, uric acid (UA), dopamine (DA), methoxymethyldiphenylamine (DPA), H_2_O_2_, naringin and naringenin as well as some common concentration ions such as K^+^, Na^+^, Ca^2+^, Hg^+^, Mg^2+^, Fe^3+^, Al^3+^, Ag^+^, Pb^2+^, Zn^2+^, Ba^2+^, Cd^2+^, Co^2+^, I^−^, IO_3_^−^, S^2−^, S_2_O_8_^2−^ and PPi. As shown in Figure 5, the fluorescence intensity of the PMNPs assay at 377 nm significantly decreased only for NRG and NRGe, demonstrating that the as-prepared PMNPs probe for NRG and NRGe possessed good selectivity. However, the probe could not distinguish between the two when both existed.

### 3.5. Fluorescence Quenching Mechanism

In general, the fluorescence quenching mechanism can take place via donor acceptor charge-transfer complexes, static/binding-related quenching and dynamic quenching [35], which occur through many processes such as fluorescence resonance energy transfer (FRET) [36,37,38,39], photoinduced electron transfer (PET) [40,41,42] and innerfilter effect (IFE) [43,44,45].

**Figure 6:**
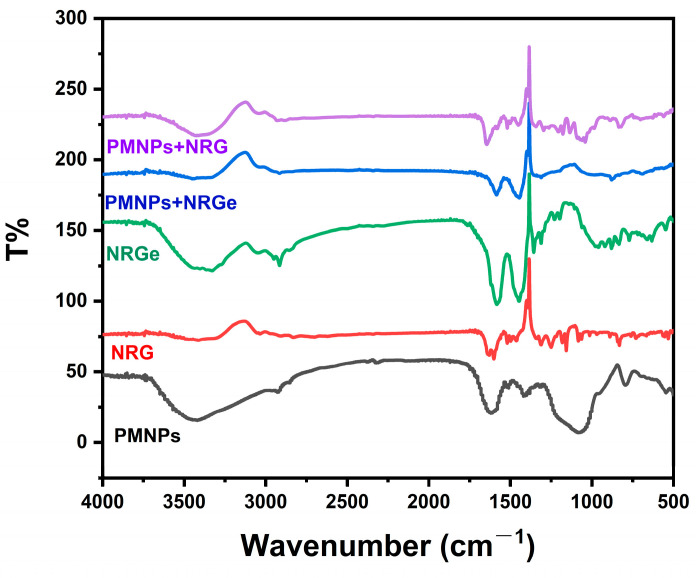
FTIR spectra of PMNPs (black line), NRG (red line), NRGe (green line), PMNPs + NRGe (blue line), PMNPs + NRG (purple line).

**Figure 7:**
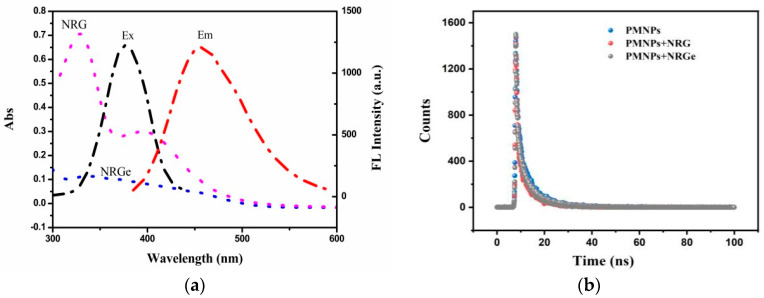
(**a**) Overlay of fluorescence spectrum of PMNPs and absorption spectrum of NRG (blue line) and NRGe (magenta line). (**b**) Time-resolved fluorescence decay profiles of PMNPs in the absence (blue ball) and presence of NRG (red ball) and NRGe (grey ball).

In order to study the quenching mechanism of the PMNPs by NRG and NRGe, the FT-IR spectra of the sensing system were first investigated. As shown in Figure 6, no obvious change was observed in the spectrum of PMNPs + NRG and PMNPs + NRGe, compared with the spectra of only PMNPs, NRG and NRGe. This result proves that no new substance was produced, demonstrating that the quenching mechanism is possibly not attributed to the interaction between the probe and the analytes and static quenching [46]. Figure 7a shows that NRGe exhibited a broad absorption at 350 nm and NRG displayed visible absorption at 330 and 390 nm. NRG showed an overlap with the excitation spectra of the PMNPs to a large extent. NRGe showed an overlap both with the excitation and emission spectra of the PMNPs. These results suggested that the quenching mechanism may be attributed to the IFE. The IFE phenomenon exists between the absorber and fluorophore if the analyte molecule possesses a strong absorption at the excitation or emission wavelength of the fluorophore [39]. To further investigate and determine the type of quenching mechanism precisely, the fluorescence lifetimes of the PMNPs with NRG/NRGe were measured, respectively, via TCSPC. Fluorescence decay was calculated based on the following equation: τ average = (B_1_τ_1_^2^ + B_2_τ_2_^2^)/(B_1_τ_1_ + B_2_τ_2_) [34]. As revealed in Figure 7b, in the presence of the quencher molecules of NRG/NRGe (4.98 and 5.10 ns), the fluorescence lifetime values of PMNPs (5.4 ns) did not exhibit any visible change (Table 3), which confirms that the quenching mechanism was the IFE process rather than the FRET process [46,47].

### 3.6. Practical Application in Real Samples

Moreover, we studied the practicality of this method for NRG and NRGe detection, respectively, in real samples including grapefruit peel and orange juice. The results are listed in Table 4. It could be observed that the average concentration of NRG and NRGe in the grapefruit peel sample was estimated to be 12.40 and 8.26 μM, respectively.

The average concentrations of NRG /NRGe in the orange juice sample were measured to be 1.23 and 2.63 μM, respectively. The nanosensor showed good recovery percentages between 92% and 103% and all relative standard deviations (RSD) were below 4.6%. These experimental results show that PMNPs used as a fluorescent probe show reproducibility and accuracy and have great potential for applications in NRG and NRGe assays with grapefruit peel and orange juice samples.

## 4. Conclusions

In summary, a novel fluorescent platform was developed to measure NRG and NRGe. The sensing strategy was simple and effective. The sensor achieved NRG and NRGe concentration in the range of 0–200 μM with limits of detection of 0.33 and 0.83 μΜ, respectively. This developed PMNP-based fluorescent probe for NRG and NRGe determination is simple, fast and efficient. The fluorescence quenching mechanism of PMNPs toward the NRG and NRGe was found to be the IFE. Moreover, the sensor was successfully applied to the detection of NRG and NRGe in different types of citrus samples.

## Figures and Tables

**Table 1:** Comparison of methods for NRG determination.

Method	Probe	Linear Range	LOD	Reference
HPLC	-	-	0.2 μmol/L (LOD)	[17]
Electrochemical Method	-	-	0.55 μmol/L (LOD)	[25]
Photo-electrochemistry	CPPI-TiO_2_/Cd_S_/FTO	1~332 μmol/L	0.03 μmol/L (LOD)	[21]
Electrochemistry Method	Hanging mercury drop electrode	0.172~68.8 μmol/L	0.05 μmol/L (LOD)	[22]
Fluorescence method	PMNPs	1~200 μmol/L	0.33 μmol/L (LOD)	[This work]

**Table 2:** Comparison of methods for NGRe determination.

Method	Probe	Linear Range	LOD	Reference
HPLC	-	-	1.286 μmol/L (LOD)	[17]
Electrochemical method	- rGO/GSH/GCE	10~1200 μmol/L	3.33 μmol/L (LOD)	[24]
Fluorescence method	PMNPS	1~200 μmol/L	0.83 μmol/L(LOD)	[This work]

**Table 3:** Time-resolved fluorescence decay of PMNPs in the absence and presence of NRG /NRGe.

Samples	τ_1_ (ns)	τ_2_ (ns)	B_1_	B_2_	τ_avg_ (ns)
PMNPs	0.9191	6.5754	975.2305	519.9597	5.4
PMNPs + NRG	0.6949	6.1699	1081.1244	417.5455	4.93
PMNPs + NRGe	0.8548	6.4076	1042.3488	454.1409	5.10

**Table 4:** Analytical results of NRG and NRGe in real samples using the PMNP-based fluorescence method.

Samples (%)		Detected (μΜ)	Added (μΜ)	Found (μΜ)	Recovery (%)	RSD
Grapefruit peel	NRG	12.4	2.5	14.85	102.0	3.4
			5.0	17.55	103.0	4.6
			10.0	22.48	99.2	4.3
	NRGe	8.26	5.0	13.28	100.4	4.4
			10.0	18.16	99.0	3.2
			20.0	27.10	94.2	4.5
Orange juice	NRG	1.23	2.5	3.78	102.0	2.4
			5.0	6.16	98.6	1.8
			10.0	11.19	99.6	1.5
	NRGe	2.63	5.0	7.52	97.8	3.2
			10.0	12.42	97.9	4.2
			20.0	21.03	92.0	3.8

## Data Availability

The original contributions presented in the study are included in the article, further inquiries can be directed to the corresponding author.

## Supplementary Materials

The following supporting information can be downloaded at: https://www.mdpi.com/article/10.3390/ma17163919/s1, Figure S1: The size distribution of the prepared PMNPs; Figure S2: UV-vis absorption spectra of L-Met and PMNPs.
