# Supplementary material for: A Polymethionine Nanoparticle Fluorescent Probe for Sensitive Detection of Naringin and Naringenin
Source: Materials (Basel). 2024 Aug 7;17(16):3919. doi: 10.3390/ma17163919 (PMC11355485; doi:10.3390/ma17163919)
Supplement: Supplementary file 1 [file materials-17-03919-s001.zip › materials-3117483-supplementary.pdf]

Article

A Polymethionine Nanoparticle Fluorescent Probe for Sensitive Detection of Naringin and Naringenin

Yuhong Jiao [1], Lu Li [1,*], Jinlong Ge [1], Yanfang Tai [1] and Hui Han [2]

[1] School of Materials and Chemical Engineering, Bengbu University, Bengbu 233000, China; jwh@bbc.edu.cn (Y.J.); jinlongge2005@126.com (J.G.); taiyanfang@163.com (Y.T.)
[2] Anhui Triumph Applied Materials Co., Ltd., Bengbu 233000, China; 13956386350@163.com
* Correspondence: lulihbnu@163.com

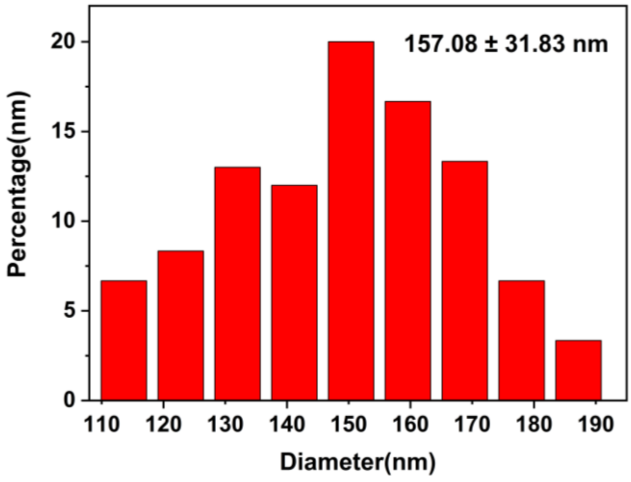

Figure S1. The size distribution of the prepared PMNPs.

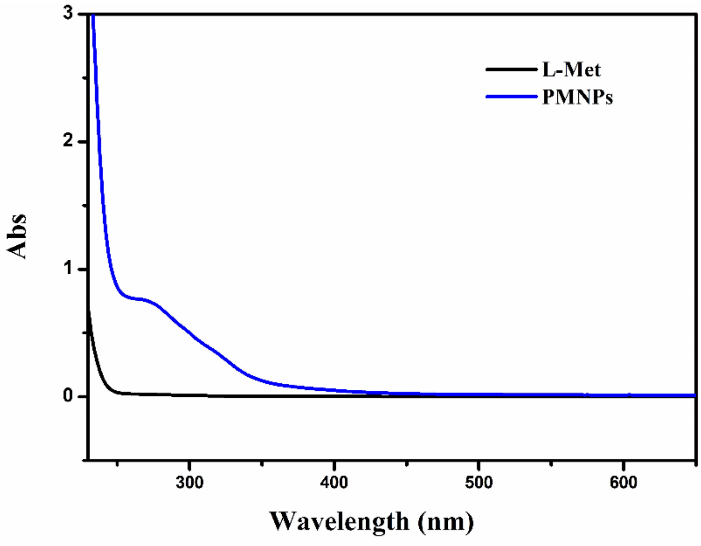

Figure S2. UV-vis absorption spectra of L-Met and PMNPs.
